# Lessons learned from HIV can inform our approach to COVID‐19 stigma

**DOI:** 10.1002/jia2.25504

**Published:** 2020-05-04

**Authors:** Carmen H Logie

**Affiliations:** ^1^ Factor‐Inwentash Faculty of Social Work University of Toronto Toronto Canada

**Keywords:** COVID‐19, stigma, HIV, gender, community, history

Stigma targeting people associated with COVID‐19, particularly persons of Asian descent, has been reported in media spanning diverse global contexts [1]. The United Nations described that “fear, rumours and stigma” are key challenges accompanying COVID‐19 [2]. The convergence of its framing as a “foreign virus” and an “infodemic” ignited this fear and stigma [2, 3]. This is not a new phenomenon. Blaming epidemics on a foreign “other” is a recurring historic narrative [4, 5]. We can leverage our four decades of HIV research to understand and address COVID‐19 stigma.

First, HIV reflects the health, moral and racial dimensions of stigma theorized by Goffman [6] and aligned with historical patterns of disease attribution [4, 5]. Stigma is produced in social processes of labelling that differentiate persons characterized as “normal” from the “abnormal” other. Archetypes of the other include racial and religious minorities as well as people labelled as physically unhealthy or “immoral” [6]. In the early 1980s the HIV epidemic – initially coined Gay‐Related Immune Deficiency (GRID) – was conceptualized as a plague that impacted “at risk” populations in the US known as the “4‐H’s” (haemophiliacs, heroin users, homosexuals, Haitians) [7, 8]. This framing blamed racial (Haitian) and “immoral” (e.g. gay men) others and positioned a foreign location (Haiti) as the origin of HIV in the US. The World Health Organization deliberately named COVID‐19 to avoid conflation with a location of origin [3], yet referrals to it as the “Chinese” and “Wuhan” virus persist [9]. The arrests of people for breaching COVID‐19 public health measures [10] – and subsequent labelling as “intentional murderers” [11] and “super spreaders” [12] – signal the creation of the “immoral” other. These arrests contradict UNAIDS recommendations to avoid criminal repercussions for breaching COVID‐19 public health restrictions [13]. Similar to HIV, we need to address several facets of COVID‐19 stigma to effectively reduce it. These include exposing and eliminating racism and xenophobia and recognizing the social processes of othering already experienced by persons blamed for COVID‐19 (including stigma and socio‐economic exclusion experienced by immigrants [14]).

Second, HIV has taught us about the complexity of stigma. We are moving away from siloed stigma research on individual health conditions (e.g. HIV, mental health), social identities (e.g. race, sexual orientation) and practices (e.g., sex work, drug use) [15]. Instead, stigma is understood as intersectional, social ecological, and produced by drivers (e.g., misinformation) and facilitators (e.g., inequitable social norms) [15, 16, 17, 18]. Intersecting stigma – such as racism and poverty – interact with HIV‐related stigma to harm health engagement and outcomes [16, 17] and may present analogous barriers to COVID‐19 testing and treatment [14].

Stigma also operates across multiple, interacting dimensions of life. Social ecological approaches to HIV remind us that stigma is intrapersonal (affecting our self‐perception and mental health), interpersonal (altering our relationships), social (embedded in community norms and values) and structural (reproduced institutionally in health, legal, employment and other practices) [15, 16]. Researchers can apply this lens to explore COVID‐19 stigma’s effects on mental health, intimate relationships [18], community cohesiveness, and interactions with police, employers, healthcare providers, among others.

Stigma experiences are shaped by intersecting social identities. Researchers have called for a gender‐based analysis of COVID‐19 [18, 19]. We urgently need to examine the gendered nature of COVID‐19 stigma, particularly in light of HIV‐related stigma research that shows its associations with gender‐based violence [e.g. 20]. Age is another identity that may shape COVID‐19 stigma manifestations. There are complex associations between HIV‐related stigma and age, whereby older persons living with HIV may experience reduced health effects of stigma [21, 22]. This could differ from COVID‐19, where the distressing choice of rationing intensive care hospital beds and ventilators has sparked debate over choosing who should live and who should die [23, 24]. The recommended utilitarian approach favours prioritizing treatment for young, severely ill persons [24]. What implications does this scarcity of COVID‐19 medical resources have on stigma towards older persons? Understanding specific contexts of COVID‐19 stigma can inform tailored mitigation strategies. However, the great challenge remains that COVID‐19 is a moving target with continually changing dynamics. Groups impacted by stigma may change as the pandemic evolves. While Asian communities were initially blamed for COVID‐19 [1, 9], will this liability shift to other marginalized communities, such as undocumented immigrants, homeless persons, and others who experience barriers to testing and care [14]?

We can also apply lessons from HIV‐related stigma reduction interventions to COVID‐19. Community‐based approaches to reducing HIV‐related stigma focus on generating solidarity and reclaiming identities [3, 13, 16, 25]. Such COVID‐19 stigma resistance tactics have already emerged, evidenced with the Twitter hashtags #IamNotaVirus, #NoSoyUnVirus and #JeNeSuisPasUnVirus. There is a rich evidence‐base of HIV‐related stigma interventions for healthcare providers that provide HIV information, share how stigma affects communities, encourage reflection on personal biases and ensure institutional support for stigma mitigation [13, 26, 27]. Other strategies include participatory learning through engaging activities such as discussions, games and role‐play [26, 27]. The contact approach involves people who have experienced the stigma being targeted (e.g. persons living with HIV, persons experiencing COVID‐19 stigma) delivering the intervention to provide a face to the pandemic that in turn can foster empathy and reduce othering [26, 27].

Moving forward we need not only focus on the stories of hardship in the midst of an epidemic [28], but to also remember the complexity and fullness of people’s lives. For instance, there are videos circulating on social media of people quarantined in Italy for COVID‐19 singing to one another from their balconies. The HIV epidemic simultaneously produced stigma and created communities among affected persons [7, 8]. Creating space for stories of COVID‐19 that reveal stigma *and* solidarity, of front‐line healthcare workers’ experiences, and of people living in quarantine, can reduce fear and spark empathy by helping us to see ourselves and our communities reflected in the pandemic [28]. Understanding our shared humanity and the precarity of distinguishing the “sick” and “healthy” may be a step towards fostering solidarity. Sontag [4] reminds us that we are interconnected in our vulnerability to sickness:

Illness is the night‐side of life, a more onerous citizenship. Everyone who is born holds dual citizenship, in the kingdom of the well and in the kingdom of the sick. Although we all prefer to use only the good passport, sooner or later each of us is obliged, at least for a spell, to identify ourselves as citizens of that other place. (p. 3).

## Competing interest

None.

## Author’s contributions

CHL conceptualized and wrote the manuscript. She read and approved the final manuscript.
